# Cytoadhesion to gC1qR through *Plasmodium falciparum* Erythrocyte Membrane Protein 1 in Severe Malaria

**DOI:** 10.1371/journal.ppat.1006011

**Published:** 2016-11-11

**Authors:** Ariel Magallón-Tejada, Sónia Machevo, Pau Cisteró, Thomas Lavstsen, Pedro Aide, Mercedes Rubio, Alfons Jiménez, Louise Turner, Aida Valmaseda, Himanshu Gupta, Briegel De Las Salas, Inacio Mandomando, Christian W. Wang, Jens E. V. Petersen, Jose Muñoz, Joaquim Gascón, Eusebio Macete, Pedro L. Alonso, Chetan E. Chitnis, Quique Bassat, Alfredo Mayor

**Affiliations:** 1 ISGlobal, Barcelona Ctr. Int. Health Res. (CRESIB), Hospital Clínic—Universitat de Barcelona, Barcelona, Spain; 2 Centro de Investigação em Saúde da Manhiça, Maputo, Mozambique; 3 Centre for Medical Parasitology, Department of International Health, Immunology, and Microbiology, Faculty of Health Sciences, University of Copenhagen, and Department of Infectious Diseases, Copenhagen University Hospital (Rigshospitalet), Copenhagen, Denmark; 4 Consorcio de Investigación Biomédica y Salud Pública, Madrid, Spain; 5 Facultad de Medicina, Universidad de Antioquia, Medellín, Colombia; 6 Department of Parasites and Insect Vectors, Institut Pasteur, Paris, France; 7 Malaria Group, International Centre for Genetic Engineering and Biotechnology, New Delhi, India; 8 ICREA, Pg. Lluís Companys 23, 08010 Barcelona, Spain; MRC National Institute for Medical Research, UNITED KINGDOM

## Abstract

Cytoadhesion of *Plasmodium falciparum* infected erythrocytes to gC1qR has been associated with severe malaria, but the parasite ligand involved is currently unknown. To assess if binding to gC1qR is mediated through the *P*. *falciparum* erythrocyte membrane protein 1 (PfEMP1) family, we analyzed by static binding assays and qPCR the cytoadhesion and *var* gene transcriptional profile of 86 *P*. *falciparum* isolates from Mozambican children with severe and uncomplicated malaria, as well as of a *P*. *falciparum* 3D7 line selected for binding to gC1qR (*Pf*3D7^gC1qR^). Transcript levels of DC8 correlated positively with cytoadhesion to gC1qR (rho = 0.287, *P* = 0.007), were higher in isolates from children with severe anemia than with uncomplicated malaria, as well as in isolates from Europeans presenting a first episode of malaria (n = 21) than Mozambican adults (n = 25), and were associated with an increased IgG recognition of infected erythrocytes by flow cytometry. *Pf*3D7^gC1qR^ overexpressed the DC8 type PFD0020c (5.3-fold transcript levels relative to Seryl-tRNA-synthetase gene) compared to the unselected line (0.001-fold). DBLβ12 from PFD0020c bound to gC1qR in ELISA-based binding assays and polyclonal antibodies against this domain were able to inhibit binding to gC1qR of *Pf*3D7^gC1qR^ and four Mozambican *P*. *falciparum* isolates by 50%. Our results show that DC8-type PfEMP1s mediate binding to gC1qR through conserved surface epitopes in DBLβ12 domain which can be inhibited by strain-transcending functional antibodies. This study supports a key role for gC1qR in malaria-associated endovascular pathogenesis and suggests the feasibility of designing interventions against severe malaria targeting this specific interaction.

## Author Summary

*Plasmodium falciparum* sequesters in vital organs. This phenomenon mediated by cytoadhesion of infected-erythrocytes to host receptors in the microvasculature, contributes to the development of severe malaria. Although cytoadhesion to Endothelial Protein-C Receptor has a central role in severe malaria, other host receptors are also likely to be involved. Our results generated by the analysis of *P*. *falciparum* isolates from Mozambican patients and laboratory parasite lines indicate that a specific domain (DBLβ12) from DC8-type PfEMP1s can bind to the human receptor gC1qR, previously associated with severe malaria. Our findings revealed that antibodies against PfEMP1 could provide strain-transcending inhibition of gC1qR-binding. Overall, these results support a key role for the adhesion to gC1qR in malaria-associated endovascular pathogenesis and the feasibility of new interventions targeting this specific interaction.

## Introduction

Case fatality rates for severe malaria (SM) remain unacceptably high even after administration of effective anti-malarial drugs [1]. There is an urgent need to develop novel interventions against life-threatening malaria. However, the mechanisms underlying the clinical heterogeneity and spectrum of malaria [2] remain largely unknown. The general state of health and physiological condition of the host, in particular variations in host immunity, together with genetic predisposition and parasite factors involved in the virulence of the infection, might influence the progression of malaria towards a life-threatening outcome. Sequestration of infected erythrocytes (IE) in vital organs is believed to constitute a key pathogenic event in *P*. *falciparum* SM [3], eventually leading to hemorrhages, thrombi formation and pathological inflammation [4], all at the basis of microvascular obstruction [4–6]. Strategies to inhibit or prevent parasite sequestration thus have the potential to reduce the high fatality rate in SM.

Surface proteins at the interface of malaria parasites and the human host contribute to sequestration through the cytoadhesion of IEs to the vascular endothelium, to uninfected erythrocytes to form rosettes [7] and to IEs through platelet binding to form agglutinates (Platelet-mediated [PM]-agglutination) [8]. Cytoadhesion is primarily mediated by interactions between *Plasmodium falciparum* erythrocyte membrane protein 1 (PfEMP1) [9] and host receptors such as CD36 [10], ICAM-1 [11], CSA [12], heparin [7], EPCR [13] and gC1qR [8,14]. PfEMP1 is a family of highly diverse antigens located on the surface of mature stage IEs that contain 2–9 adhesion domains termed DBL (Duffy binding-like) and CIDR (cysteine-rich interdomain region). Each parasite contains ∼60 different *var* genes per haploid genome that encode PfEMP1s, which subvert acquisition of protective immunity [15] through constant transcriptional switching [16] and mutually exclusive expression [17]. Antibodies to PfEMP1 that occur after natural infections or after immunization with recombinant PfEMP1 domains are predominantly variant- and strain-specific, as expected for highly variable parasite antigens [18–20]. However, epidemiological observations that children acquire immunity to non-cerebral severe malaria after a small number of infections [21] suggest that strain-transcending antibodies recognizing conserved epitopes on PfEMP1 may occur [19,22], or that the parasites that cause severe malaria are of restricted antigenic types [23,24].

PfEMP1s can be classified into three major groups (A, B and C) and two intermediate groups (B/A and B/C), based on motifs in non-coding sequences and locus position [25]. Whereas most group B and C PfEMP1 proteins appear to be under selection to bind CD36 [26] and tend to be associated with uncomplicated and asymptomatic malaria [27,28], groups A and B/A are often expressed in young children with limited malaria immunity [23] and in those with SM [28–31]. A subset of these A and B/A PfEMP1 variants that contain a combination of adhesion domains, termed domain cassettes 8 and 13 (DC8 and DC13) [32–34], can bind through their CIDRα1.1/4/5/7 domains to Endothelial Protein C Receptor (EPCR) [13]. It has been suggested that EPCR-mediated parasite cytoadhesion could interfere with activation of cytoprotective and anti-inflammatory pathways, which in turn may contribute to severe malaria pathology [13]. However, adhesion to human cell lines is likely to be mediated by interaction with several receptors [35]. Indeed other domains of DC8 and DC13 PfEMP1 variants have been shown to bind avidly to endothelial cells from different tissues through unknown host receptors [36]. These data highlight the heterogeneity of receptors used by IEs in different vascular beds and the importance of identifying other receptors involved in host-parasite interactions.

*P*. *falciparum* IEs use gC1qR as a receptor for both cytoadhesion to human cells and platelet-mediated clumping [14], a cytoadhesion phenotype which has been associated with SM in Mozambican children [8]. Human gC1qR is a multi-functional cellular protein expressed on a wide range of tissues and cell types including endothelial cells, lymphocytes, dendritic cells and platelets [37]. In addition to modulating the activation of complement through binding to C1q [38], gC1qR can serve as a receptor for diverse pro-inflammatory ligands [39] and functional antigens of viral and bacterial origin to promote pathogen attachment and/or entry [40]. However, the protein used by malaria parasites to mediate cytoadhesion of IEs to gC1qR is currently unknown.

Selection of IT/FCR3 parasite lines for binding to human brain microvascular endothelial cells (HBMEC) was associated with an up-regulation of DC8- and DC13-PfEMP1 and an increase in binding to gC1qR [33]. Based on this observation, we hypothesized that PfEMP1s containing DC combinations associated with SM may mediate binding to gC1qR. To address this, we assessed the *var* expression patterns and gC1qR cytoadhesion profile of *P*. *falciparum* isolates collected from Mozambican children [8] and in a *P*. *falciparum* 3D7 line selected *in vitro* for binding to gC1qR. The relationship of *var* transcript levels with disease severity, previous malaria exposure and antibody-mediated recognition of IEs was also analyzed. Our results demonstrate that transcript abundance of DC8 in field isolates is associated with binding of IEs to gC1qR and that DBLβ12 from the DC8-type PFD0020c mediates such interaction in the *P*. *falciparum* 3D7 line selected for binding to gC1qR. The successful induction of strain-transcending antibodies against DBLβ12 with activity to inhibit binding to gC1qR by field isolates suggests shared surface epitopes amongst heterologous gC1qR-binding PfEMP1 variants and the feasibility to designing interventions to prevent severe malaria.

## Results

### Study population and clinical outcomes

Blood samples from 132 malaria patients were used in the study, 111 from Manhiça, Mozambique (86 children and 25 adults) and 21 from European travelers (Table 1). Among the Mozambican children, 43 had uncomplicated malaria (UM) and 43 had SM, defined as severe anemia, acidosis or respiratory distress, multiple seizures, prostration, cerebral malaria or hypoglycemia (Table 1) [8]. Among the 43 cases of severe malaria, 19 (44%) had a single criteria of malaria severity and the rest overlapping symptoms (13 [30%] had two and 11 [26%] three or more). Prostration was observed in 34 (79%) of the children, acidosis/respiratory distress in 17 (39%), severe anemia in 13 (30%) and multiple seizures in 11 (26%), whereas cerebral malaria and hypoglycemia was observed only in 3 and 2 of the children, respectively (Table 1). European travelers were coming from Western Africa (Ghana, Republic of Côte d'Ivoire, The Gambia, Guinea, Equatorial Guinea, Togo, Senegal and Burkina Faso), Middle Africa (Cameroon, Congo and Central African Republic) and Eastern Africa (Mozambique and Madagascar), with none of them presenting SM at recruitment. Parasitemia, quantified by qPCR, was the highest in Mozambican adults, followed by SM and UM cases, with travelers showing the lowest levels of parasitemia (*P* = 0.022). No differences were observed in the multiplicity of infection (MOI) between groups (*P* = 0.106).

**Table 1:** Characteristics of the patients with malaria included in the study

	Spain	Mozambique			
Patient characteristics	Travelers (n = 21)	SM (n = 43)	UM (n = 43)	Adults (n = 25)	*P*
Age (years), median (IQR)	34 (29–40)	2.4 (1.3–3.6)	2.6 (1.3–3.6)	36 (30–46)	0.791
qPCR Parasite density*, median(IQR)	724(322–9973)	9060(2290–31982)	3618(1050–13623)	10260(2267–38327)	0.022
MOI, median (IQR)	2 (1–3)	3 (3–5)	3 (2–4)	2 (2–3)	0.106
Males, n (%)	16 (76)	28 (65)	28 (65)	16 (64)	1.000
**Clinical manifestation of SM** ^**a**^ **(n)**					
Cerebral malaria	-	3	-	-	
Severe anaemia	-	13	-	-	
Multiple seizures	-	11	-	-	
Prostration	-	34	-	-	
Hypoglicemia	-	2	-	-	
Acidosis/Respiratory Distress	-	17	-	-	

IQR, Interquartile range; SM, severe malaria; UM, uncomplicated malaria; MOI, multiplicity of infection.

*, Expressed as parasites per μL.

^a^, Nineteen (44%) out of the 43 SM cases had a single criterion of malaria severity and the rest overlapping symptoms (13 [30%] had two and 11 [26%] three or more).

### Transcript level of DC8 and DC11 *var* genes correlate with gC1qR cytoadhesion in Mozambican isolates

**Fig 1:**
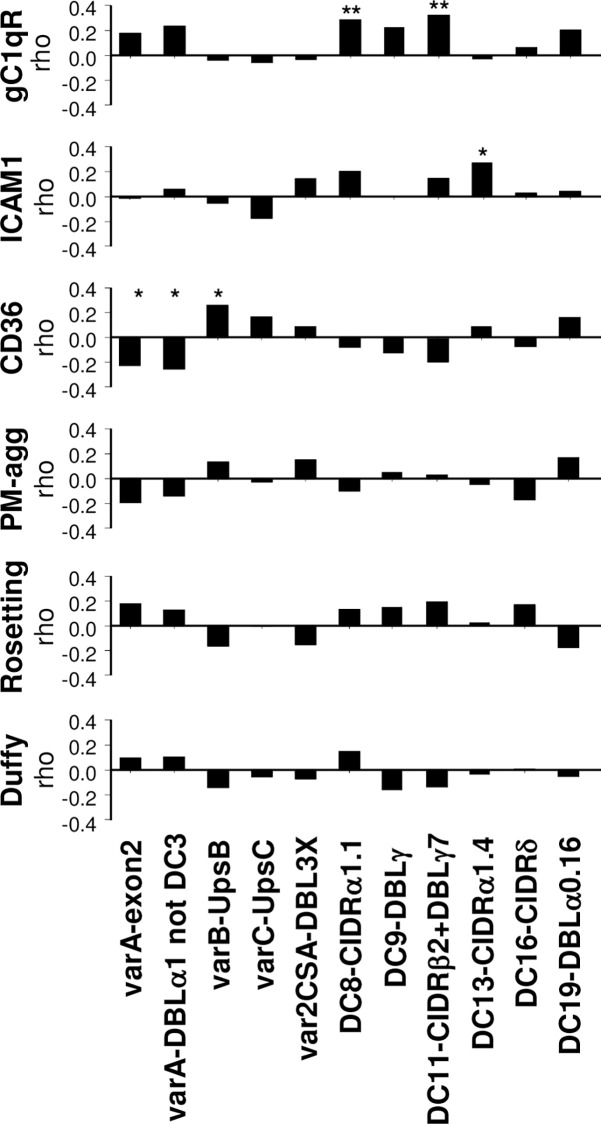
Correlation between *var* transcript levels and cytoadhesive phenotypes of *P*. *falciparum* isolates from Mozambican children. The relationship between adhesion and transcript levels of *var*/DCs was assessed by Spearman correlation analysis, with * indicating *P*<0.05 and ** if statistically significant after Benjamini-Hochberg correction for the six adhesive phenotypes tested. PM-agg: platelet-mediated agglutination.

The relationship between cytoadhesion and *var/DCs* transcript levels was assessed among *P*. *falciparum* isolates collected from Mozambican children (n = 86; Fig 1). Adhesion to CD36 was the most frequent cytoadhesion phenotype (76/86 [88%]; median binding of 180 IEs/mm^2^, IQR[101–353]), followed by PM-agglutination (57/86 [66%]; median of 7%, IQR[2–22]), adhesion to gC1qR (38/86 [44%]; median binding of 60 IEs/mm^2^, IQR[45–155]), ICAM1 (37/86 [43%]; median binding of 55 IEs/mm^2^, IQR[39–105]) and rosetting (31/86 [36%]; median of 2%, IQR[1–5]; Table A in SI Text). The percentage of isolates expressing *var/DCs* ranged from 41% (35/86) for DC13-CIDRα1.4 to 100% for *varA*-exon2, varB-UpsB and DC11-CIDRβ2+DBLγ7 (Table B in SI Text). Adhesion to gC1qR correlated positively with DC8 transcript levels (targeted by DC8-CIDRα1.1, *rho*: 0.287, *P* = 0.007) and with DC11 (*rho*: 0.324, *P* = 0.002). Adhesion to ICAM1 showed a positive correlation with transcript levels of DC13 (*rho*: 0.273, *P* = 0.011). Adhesion to CD36 correlated positively with *varB* (*rho*: 0.259, *P* = 0.016) and negatively with *varA* (*rho* = -0.228, *P* = 0.035) and *varA*-notDC3 (*rho*: -0.256, *P* = 0.018). No association was found between *var* transcript levels, PM-agglutination, rosetting or binding to the negative control Duffy receptor (Fig 1).

**Fig 2:**
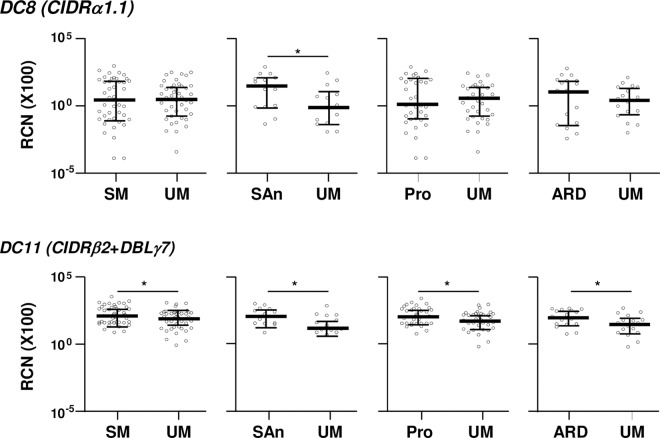
Transcript levels of DC8 and DC11 by severe malaria symptoms in Mozambican children. Transcript levels (y axis) correspond to relative copy number of target genes relative to seryl-tRNA synthetase gene copies (X100). Bars represent the median and interquartile range. Transcript levels were compared between matched case/control pairs by Sign-test, with * indicating *P*<0.05. RCN: Relative copy number; DC: Domain Cassette; SM: severe malaria; UM: uncomplicated malaria; SAn: severe anemia; Pro: prostration; ARD: acidosis or respiratory distress.

**Fig 3:**
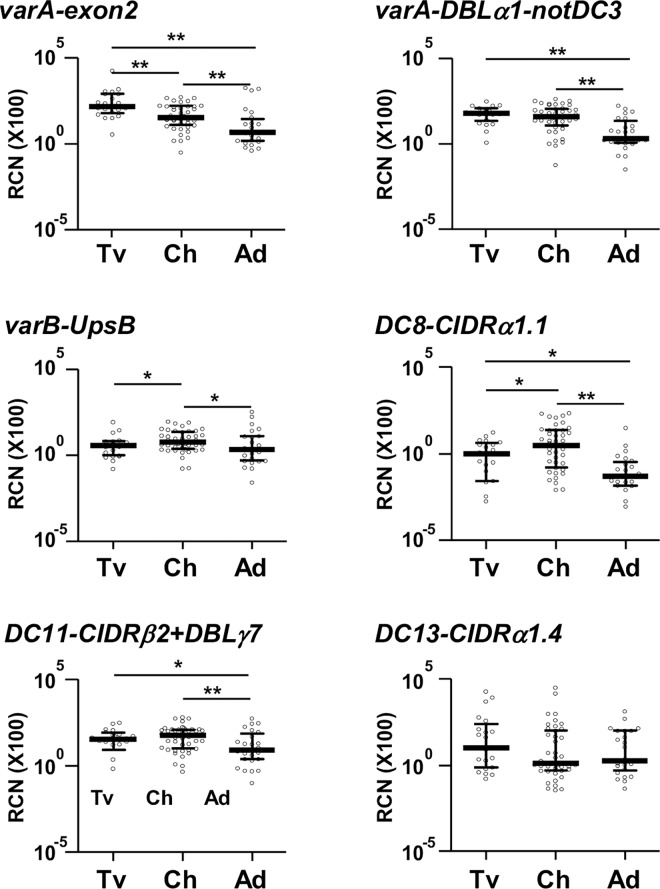
Transcript levels of *var*/DCs in *P*. *falciparum* isolates from travelers, children with uncomplicated malaria and adults. Transcript levels (y axis) correspond to relative copy number relative to seryl-tRNA synthetase gene copies (X100). Bars represent the median and interquartile range. Transcript levels were compared between groups by Mann-Whitney test, with * indicating *P*<0.05 and ** if statistically significant after Benjamini-Hockberg correction. RCN: Relative copy number; DC: Domain Cassette; Tv: travelers, Ch: Mozambican children, Ad: Mozambican adults.

DC11 transcript levels were lower in isolates from children with UM than in those from SM children (*P* = 0.043), severe anemia (*P* = 0.022), prostration (*P* = 0.050) and acidosis/respiratory distress (*P* = 0.044, Fig 2 *and* Table C in SI Text). Similarly, transcript levels of DC8 were higher in children with severe anemia compared to their UM pairs (*P* = 0.030, Fig 2). Both DCs were transcribed at similar levels by isolates from travelers and children, being the lowest in isolates from Mozambican adults (Fig 3).

### Increased IgG recognition of isolates highly transcribing DC8 *var* genes

**Fig 4:**
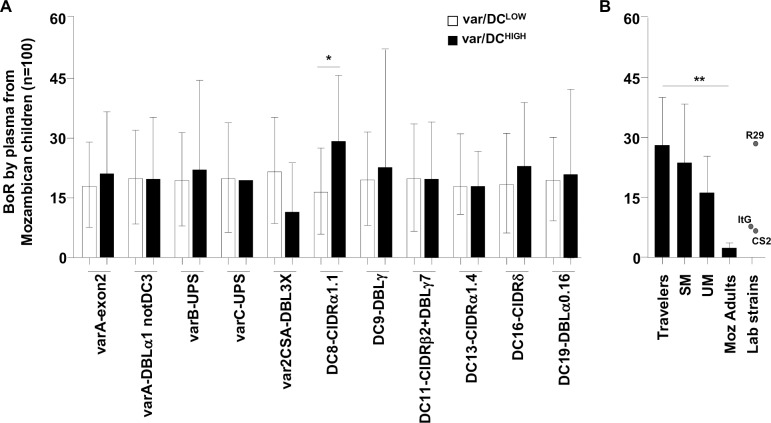
Breadth of IgG recognition of *P*. *falciparum* isolates according to *var* transcript levels and origin. Geometric Mean Fluorescence Intensity (GMFI) values from each parasite/plasma combination were scored in relation to the threshold of positivity (GMFI of negative controls plus two standard deviations), with a score of 0 assigned if GMFI values were below the cut-off; 1 if the value was between one- and two-fold the cut-off; 2 if the value was between two- and three-fold the cut-off; and so on until a maximum score of 5. Breadth of IgG recognition (BoR) was calculated as the sum of scores obtained for each parasite and expressed as percentage of the maximum score possible. BoR was compared between A) isolates transcribing *var*/DCs at low- or high- levels by negative binomial regression models adjusted by age and B) between isolates collected from travelers (n = 3), severe malaria (SM, n = 23), uncomplicated malaria (UM, n = 15) children and adults (Moz adults, n = 4) by test for trend across ordered groups. Bars represent the mean of BoR and standard deviation. * indicates *P*<0.05 and ** *P*≤0.001.

Isolates transcribing DC8 at high levels (i.e., copy number ≥0.5-fold of Seryl-tRNA-synthetase copy number) were more often recognized by plasmas from Mozambican children (n = 100; mean breadth of recognition: 29%, Standard Deviation (SD) 16) than those transcribing DC8 at low levels (16%, SD 11; incidence rate ratio = 2.3, 95% CI [1.2–4.5], *P* = 0.019; Fig 4A). No differences were observed for other DCs. Breadth of IgG recognition of parasites transcribing DC8 at high levels was higher among the Mozambican adult population (76%, SD 18) than among children (p = 0.010 by Signrank test). However, no difference was observed in the breadth among children with SM (p = 0.969). Finally, recognition by plasma from Mozambican children was the highest for IEs from travelers (mean breadth of recognition: 28%; SD 12), followed by isolates from SM (24%, SD 14) and UM (16%; SD 9), being the lowest for parasites from Mozambican adults (3%, SD 1; test for tend, *P*≤0.001; Fig 4B).

### DBLβ12 domain from the DC8-type PFD0020c in 3D7 is associated with gC1qR cytoadhesion

**Fig 5:**
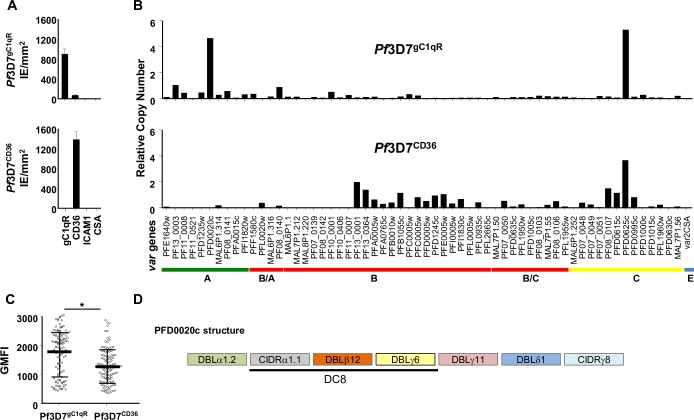
Phenotypical and molecular characterization of *Pf*3D7^gC1qR^ and *Pf*3D7^CD36.^ **A)** Binding assays of *Pf*3D7^*gC1qR*^ and *Pf*3D7^*CD36*^ over gC1qR, CD36, ICAM1 and CSA receptors. **B)** Transcriptional analysis of the *var* gene repertoire of *Pf*3D7. Transcript levels of *var* genes were determined by qPCR using primers specific for each of the *P*. *falciparum* 3D7 *var* genes and were expressed as copy number relative to the seryl-tRNA synthetase gene. **C)** Levels of IgG recognition by plasma from Mozambican children were compared by Wilcoxon matched pair test, with * indicating P≤0.001. Bars represent mean and standard deviation of geometric mean fluorescence intensity (GMFI). D) Domain structure of PFD002c.

After two rounds of *in vitro* selection for binding to gC1qR followed by a limiting dilution cloning, a *P*. *falciparum* 3D7 clone was obtained (*Pf*3D7^gC1qR^) that showed high binding to gC1qR (mean: 900 parasites/mm^2^, SD 101) and low to CD36 (71 IEs/mm^2^, SD 2. In contrast, the unselected 3D7 clone (*Pf*3D7^CD36^) showed high levels of adhesion to CD36 (1400 IEs/mm^2^, SD 159) but no adhesion to gC1qR (Fig 5A). The selection for binding to gC1qR was associated with a 1.3-fold increase in the levels of IgG recognition by plasmas from Mozambican children (Geometric Mean Fluorescence Intensity [GMFI) for *Pf*3D7^gC1qR^ of 1679, SD: 765 vs GMFI for *Pf*3D7^CD36^ of 1266, SD: 576, *P*≤0.001; Fig 5C). *P*. *falciparum* 3D7^gC1qR^ transcribed mostly the DC8-PFD0020c (4.7-fold seryl-tRNA synthetase gene), as well as PFD0625c (5.3-fold seryl-tRNA synthetase gene), whereas *Pf*3D7^CD36^ mostly expressed PFD0625c (3.7-fold seryl-tRNA synthetase gene, Fig 5B). The fold ratio of PFD0020c transcript levels in gC1qR-selected *Pf*3D7 line compared with that in unselected parasites was 4656, and 1.45 for PFD0625c.

**Fig 6:**
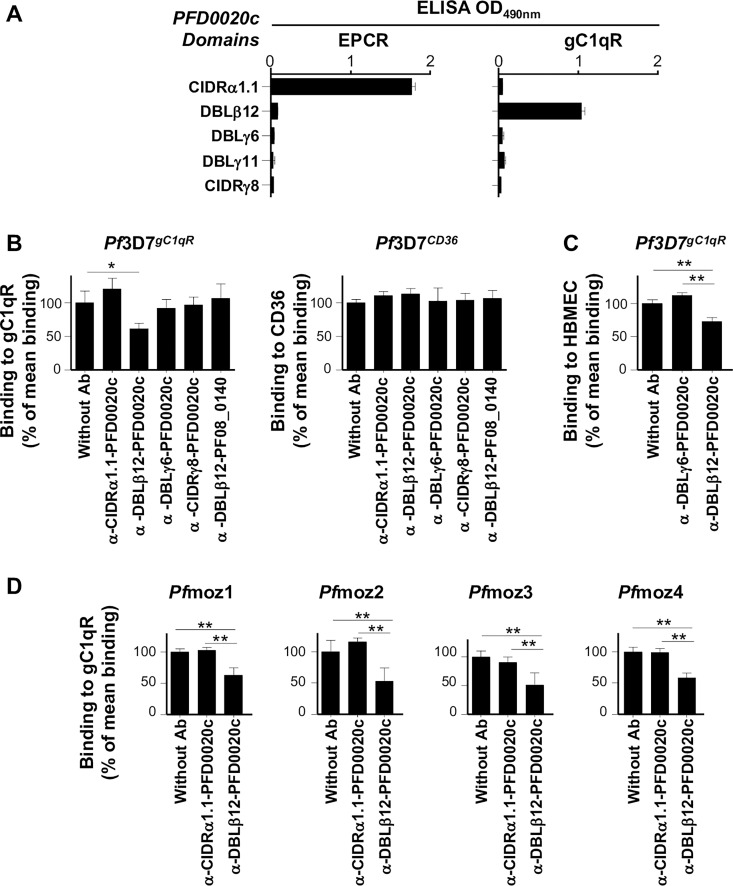
DBLβ12 domain of PFD0020c is involved in the interaction with gC1qR. **A)** Reactivity of PFD0020c domains against gC1qR and EPCR by ELISA-based binding assays. Antibody-mediated inhibition of **B)** binding to gC1qR or CD36 of *P*. *falciparum Pf3D7* ^*gC1qR*^, *Pf3D7* ^*CD36*^; **C)** binding to human brain endothelial cells (HBMEC) of *Pf3D7* ^*gC1qR*^ and **D)** binding to gC1qR of four *P*. *falciparum* Mozambican isolates. The gC1qR binding levels in absence of antibodies were 299 IEs/mm^2^ (SD 53) for *Pf*3D7^gC1qR^; 202 IEs/mm^2^ (SD 11) for *Pf*moz1; 92 IEs/mm^2^ (SD 17) for *Pf*moz2; 74 IEs/mm^2^ (SD 8) for *Pf*moz3; and 81 IEs/mm^2^ (SD 7) for *Pf*moz4. The CD36 binding level in absence of antibodies was 615 IEs/mm^2^ (SD 27) for *Pf*3D7^CD36^. Binding is expressed as the percentage of mean binding in absence of antibodies. Bars represent the mean and standard deviation. * indicates *P*<0.05 and **P<0.001.

To identify the domain(s) mediating binding to gC1qR, we assessed the ability of recombinant constructs representing extracellular domains of PFD0020c (Fig 5D) to bind to gC1qR by ELISA-binding assays. The DBLβ12 from PFD0020c, but not CIDRα1.1, DBLγ6, DBLγ11 and CIDRγ8 was shown to interact with gC1qR (Fig 6A). We further confirmed the gC1qR-binding specificity through a bead-suspension technology (Luminex) in which the beads were coupled with the seven PFD0020c domains and tested for binding to gC1qR, allowing us to confirm that only DBLβ12 was able to bind to gC1qR (Fig A in SI Text). In contrast, only CIDRα1.1 from PFD0020c reacted with rEPCR but not the other domains tested, DBLβ12, DBLγ6, DBLγ11 and CIDRγ8. Moreover, purified polyclonal IgG generated in rabbit against domain DBLβ12_PFD0020c_, at concentrations of 300 μg/mL, were able to inhibit binding of *Pf*3D7^gC1qR^ to gC1qR by ∼40% (SD 8) compared with binding of *Pf*3D7^gC1qR^ to gC1qR in absence of antibodies (*P* = 0.029). Antibodies against the other PFD0020c domains tested and antibodies against DBLβ12_PF08_0140_ did not inhibit the binding of *Pf*3D7^gC1qR^ to gC1qR (Fig 6B). IgGs against DBLβ12-PFD0020c inhibited binding of *Pf*3D7^gC1qR^ to HBMEC cells, known to express gC1qR on their surface [14], by 46% compared to the control antibody (α -DBLγ6-PFD0020c; Fig 6C). We also tested inhibition of gC1qR binding by four *P*. *falciparum* isolates collected from Mozambican children which transcribed DC8, as targeted by DC8-CIDRα1.1, or DBLβ12&DBLβ3/5 domains, at high levels (Fig B in SI Text). In all the four isolates, binding to gC1qR was also reduced by ∼50% in the presence of antibodies against DBLβ12_PFD0020c_ (Fig 6D). IgG against domain DBLβ12_PFD0020c_ did not affect binding to EPCR nor CD36 (Fig B in SI Text). Finally, we show that DBLβ12, together with DBLγ6, DBLγ11, DBLδ1 and CIDRγ8, exhibited the highest increase in IgG recognition among malaria-infected Mozambican children compared to never-exposed Spanish individuals, as well as the highest increase with age of Mozambican children (more than 2.5 years versus less than 2.5 years of age; Fig C in SI Text).

## Discussion

The combined analysis of *P*. *falciparum* isolates from malaria infected Mozambique patients and an *in vitro* selected *P*. *falciparum* 3D7 line shows a relationship between cytoadhesion to gC1qR and transcription of DC8-type *var* genes. The clinical relevance of such a phenotype has been suggested in a field study conducted in Mozambique which showed that prevalence of parasite isolates exhibiting adhesion to gC1qR was associated with multiple seizures [8], although binding levels only tended to be higher compared with isolates from children with severe malaria. In the present study, the use of primer sets targeting the most clinically-relevant DCs [28,34,41] allowed us firstly to correlate the cytoadhesion to gC1qR with abundance of DC8 *var* transcripts in Mozambican isolates. Secondly, selection of *P*. *falciparum* 3D7 line for binding to gC1qR showed the up-regulation of the DC8-PFD0020c. Recombinant DBLβ12_PFD0020c_ bound to gC1qR in ELISA assays and antibodies against this domain were able to inhibit binding of *Pf*3D7^gC1qR^ and *P*. *falciparum* Mozambican isolates to gC1qR by 50%. Overall, these results point to the DBLβ12 domain present in DC8-PfEMP1 variants as the domain that mediates cytoadhesion to gC1qR.

Cytoadhesion to gC1qR by Mozambican isolates correlated positively with their transcript levels of DC8 which, in line with previous studies [34,42,43], were higher in parasites collected from Mozambican children with severe anemia than in those with UM. Moreover, DC8 was transcribed at higher levels by isolates from individuals with limited antimalarial immunity (i.e., Mozambican children and first-time infected travelers) compared to isolates from Mozambican adults with life-long exposure to malaria. As adults and children included in this study come from the same region in Mozambique, it is unlikely that differences observed are due to spatial heterogeneities in the DC8-expressing profile of parasite populations, especially when all parasite genomes appear to have similar repertoires globally [44]. The results rather suggest an exhaustion of the *var* gene repertoire mediating cytoadhesion and severe malaria with increasing immunity. Alternatively, antigenic variants different to DC8 may increase through ectopic recombination in chronic infections [45] which are expected to be more frequent among semi-immune adults. Also, parasites transcribing DC8 at high levels were more often recognized by plasma from malaria-exposed children than parasites with low DC8 transcription. This is in line with the observation that malaria-exposed Tanzanian population acquires antibodies to EPCR-binding CIDR domains more rapidly than antibodies to other CIDR domains [46]. Although isolates highly transcribing DC8 were better recognized by plasmas from semi-immune Mozambican adults than by children, no difference was observed between plasmas from children with severe and uncomplicated malaria. This latter observation, in line with previous studies conducted in the same area [47], might be attributed to difficulties in disentangling the role of antibodies as markers of exposure and protection among infected population. Overall, these results point towards the contribution of DC8 to gC1qR binding and severe malaria, the antigenic conservation of these PfEMP1 variants, their preferential transcription by malaria parasites infecting individuals who have still not developed antimalarial immunity [34,48,49] and the need to perform longitudinal studies to assess the role of antibodies against DC8 in reducing the risk of severe malaria.

Selection of *P*. *falciparum* 3D7 line for binding to gC1qR [14] was accompanied by a marked increase in the expression of a single *varA* gene, PFD0020c, whose transcript levels were 4656-fold higher than in the unselected line, as well as by an increase in the IgG recognition of IEs by plasmas from exposed children. In contrast, the unselected line, which bound to CD36 in static assays, transcribed B and C *var* genes at relatively low levels. Similarly to previous *in vitro* studies with *P*. *falciparum* 3D7 [50,51], PFD0625c was also detected in the selected and unselected 3D7 line, which may be due to some degree of relaxed transcription in 3D7 [52]. The PFD0020c specifically up-regulated in *Pf*3D7^*gC1qR*^ is a PfEMP1 variant characterized by having three of the four domains usually found in DC8 (CIDRα1.1, DBLβ12, DBLγ4/6), differing only in the first DBLα domain. We were not able to show binding of *Pf*3D7^*gC1qR*^ to recombinant EPCR, as would have been expected by the expression of a DC8-containing *var* gene, although we did not assess this binding specificity to endothelial cells [13]. The up-regulation of a *var* gene containing DC8 after selection of *P*. *falciparum* 3D7 for gC1qR binding fits well with previous *in vitro* studies showing the transcription of PFD0020c’s orthologs, IT4var19/IT4var07 and HB3var03, after selecting parasites lines IT4 and HB3 for binding to HBMEC cells [32,33]. Importantly, up-regulation of the PFD0020c ortholog IT4var19 after selection of IT for binding to HBMEC was associated with an increased binding to gC1qR as observed in static assays [33].

Recombinant DBLβ12 from PFD0020c, but not other domains from PFD0020c (DBLα1.2, CIDRα1.1, DBLγ4/6, DBLδ1 and CIDRγ8) and from the *var* type 3 PFI1820w (consisting in domains DBLα1.3-DBLε8), showed binding to gC1qR in ELISA- and Luminex-based binding assays. Moreover, antibodies against DBLβ12 from PFD0020c were able to inhibit the binding to gC1qR by ∼40% at antibody concentrations of 300 μg/mL. This inhibition was not observed in the CD36-binder *Pf*3D7^CD36^, which suggest that DBLβ12 is the domain with the ability to bind gC1qR. DBLβ12, which consists in 149 aa and 19 homologous blocks, is present in 12 of the 399 PfEMP1s present in the genomes of seven *P*. *falciparum* laboratory strains [44], 9 of them belonging to DC8-PfEMP1 and sharing 56% of similarity at the amino acid level. DBLβ12 was shown to be among the PFD0020c domains most immunogenic in natural infections, as shown by the increase in IgG recognition by malaria-infected Mozambican children compared to never-exposed Spanish individuals, as well as the increase in IgG levels with age of Mozambican children. Importantly, antibodies against DBLβ12 from PFD0020c raised in animal models were able to inhibit binding of Pf3D7_gC1qR_ to HBMEC cells by 46% compared to the control antibody (antibodies against DBLγ6-PFD0020c), demonstrating the gC1qR-dependent adhesion of IEs to endothelial cells through the DBLβ12 domain. Finally, polyclonal antibodies against DBLβ12 from PFD0020c showed cross-inhibitory activity against all the 4 Mozambique clinical isolates sharing the same gC1qR adhesion *in vitro*, reduced binding by 50%. Three of the four Mozambican field isolates analyzed transcribed DC8, as targeted by DC8-CIDRα1.1. However, one of the isolates (Pfmoz2) did not transcribe DC8, but transcribed DBLβ12&DBLβ3/5 at high levels, suggesting that DBLβ12-containing DC8-like PfEMP1s may share the ability to bind gC1qR. Overall, these data show that parasites with a virulence-associated adhesion phenotype such as gC1qR share PfEMP1 epitopes that can be targeted by strain-transcending functional antibodies to PfEMP1. The existence of shared surface epitopes amongst functionally similar disease-associated *P*. *falciparum* parasite isolates suggests the feasibility of developing therapeutic interventions against severe malaria

This study also shows that binding level of IEs to CD36 correlated positively with transcript levels of group B genes and negatively with *varA* levels, confirming the earlier findings that parasite ligands for CD36 are PfEMP1 variants encoded by *var* genes belonging to groups B and C [26,53,54]. In contrast to other studies showing up-regulation of DC13 in children with SM [34,42], this DC was not found associated with SM in children in our study, probably due to the low prevalence (7%) of cerebral malaria in the study population. However, transcript levels of DC13 were positively correlated with binding levels to ICAM1. Although DC13 does not have a conserved DBLβ domain with a proven ICAM-1 binding capability [55] most of DC13s are flanked by DBLβ domains, and thus this DC might be associated with a ICAM1-binding DBLβ domain type yet to be described. In fact, the DBLβ domain following DC13 in PF11_0521 [44] has been shown to bind ICAM1 [56]. Importantly, results of this study provide evidences of the potential involvement of DC11 in the pathophysiology of severe malaria. DC11 transcripts were found at higher levels in parasites collected from children with SM than UM as well as in isolates from Mozambican children and first exposed individuals (travelers) compared to isolates from Mozambican adults with life-long exposure to malaria. This DC11 has been involved in rosetting mediated by IgM [57], which has been suggested as the most clinically important rosetting phenotype [58]. However, transcript levels of DC11 were not associated with rosetting in the Mozambican isolates tested. Similarly, platelet-mediated agglutination, previously associated with binding to gC1qR [14], did not show correlation with any *var* DC. These results suggest that other receptors may be involved in IE rosetting and platelet-mediated agglutination and point towards the relevance of DC11 in the physiopathology of SM. However, further work will be necessary to elucidate the role of DC11 in the severity of the malaria disease.

This study has several limitations. First, more than half of the 43 children with severe malaria included in this study (n = 24; 56%) had two or more criteria for malaria severity [59]. Such a high degree of overlap in severe symptoms, which is otherwise common in endemic areas [60], together with the limited sample size of the cerebral malaria group, may have hampered the identification of molecular correlates that are particular to a clinical form of SM. Second, the degenerate primers used in qPCR assays have incomplete coverage of the global *var* gene repertoire. Moreover, the parasite populations obtained from peripheral blood may only partially represent the sequestering parasite population. Third, the conditions of the binding assay may not allow for 100% inhibition as has been shown for other receptors [32,61]. Alternatively, residual binding may be supported by other domains, for example those present in DC11, that may also mediate gC1qR adhesion. Fourth, given limited amounts of RNA and cryopreserved IEs available from *P*. *falciparum* field isolates included in the study, we focused the transcriptional analysis on domain cassettes previously associated with severe malaria [34,57] and the binding phenotypes on those receptors previously analyzed [8]. Fifth, gC1qR binding assays were performed on five of the 7 PFD0020c domains, but we did not test multiple domains constructs potentially involved in the binding phenotype. Finally, the fact that levels of transcripts encoding certain PfEMP1 domains types associates with the cognate parasites receptor binding capability does not necessarily mean that the particular domain mediates that receptor-binding, and other PfEMP1 domains or structures (i.e., DC11) could convey parasites this binding phenotype. More studies are needed to assess the relationship between expression of DC11, binding to gC1qR and malaria severity.

In summary, the positive correlation between gC1qR cytoadhesion by *P*. *falciparum* field isolates and their DC8 transcript levels, the overexpression of DC8-PFD0020c after selection of *P*. *falciparum* 3D7 for binding to gC1qR, and the inhibition of gC1qR binding by antibodies against DBLβ12_PFD0020c_, supports that DC8-PfEMP1s mediate binding to gC1qR through a conserved motif present in the DBLβ12 domain. Overall, our findings suggest that binding to gC1qR, mediated by interactions with DBLβ12, constitutes one of the three different host receptors suggested by protease-treatment assays of IT4 [36]. Moreover, the successful induction of strain-transcending antibodies against DBLβ12 domain from the PfEMP1 variant PFD0020c capable of inhibiting binding to gC1qR by field isolates suggests shared surface epitopes amongst heterologous gC1qR-binding PfEMP1 variants and the feasibility of designing interventions to prevent severe malaria. DC8 may thus facilitate binding to endothelial cells [32,33] via the interactions with gC1qR, known to be expressed in a wide range of human cells [14], in concert with binding to EPCR [13]. Further studies are needed to assess the relationship between DC8 expression, EPCR and gC1qR cytoadhesion, and their influence on malaria disease. Similarly to EPCR, gC1qR has been implicated in inflammatory processes such as the modulation of the complement cascade [40] and suggested to mediate bacterial cell adhesion to sites of vascular injury and thrombosis [62]. Moreover, up-regulation of gC1qR in bone marrow endothelial cells through inflammatory mediators [63] could contribute to sequestration of asexual late stages observed in *ex vivo* studies [64,65]. The results of this study support the possibility of a role for gC1qR in malaria-associated endovascular pathogenesis.

## Methods

### Study population

The study was conducted at the Manhiça District Hospital (MDH) in Southern Mozambique, a malaria endemic area where transmission of *P*. *falciparum* is perennial with some seasonality and moderate intensity [66], and at the Tropical Medicine Unit in Hospital Clinic of Barcelona (HCB), Spain. Between April and November 2006, 86 children 1 to 5 years of age [8] were recruited at MDH with *P*. *falciparum* clinical malaria, defined as the presence of fever (axillary temperature ≥37.5°C) and an asexual parasitemia of *P*. *falciparum* ≥500 parasites/μL on thin blood film examination [67]. Children with SM were those presenting with at least one of the following clinical definitions: cerebral malaria, severe anemia, acidosis or respiratory distress, prostration, hypoglycemia or multiple seizures [8]. Children with clinical malaria not showing any of the mentioned signs of severity and able to take oral medication (uncomplicated malaria; UM) were sex and age (+/-3 months) matched to SM cases. All cases and controls were reviewed by the study pediatrician to confirm that malaria was the sole or principal cause of the disease. Children with concomitant positive bacteremia were excluded from the study. Non-pregnant Mozambican adults (women and men) with life-long exposure to *P*. *falciparum* (n = 25) presenting clinical malaria at MDH were recruited between 2004 and 2005 [41]. European adults presenting a first episode of malaria after a travel to malaria endemic areas (n = 21), were recruited between 2005 and 2009 at HCB (Spain) [68]. Before treatment, peripheral blood was collected by venipuncture and 2 drops were spotted onto filter paper. Following centrifugation, plasma and 300 μL of the red blood cell pellet resuspended in 3 mL of Trizol reagent (Invitrogen) were stored at -80°C. The remaining red blood cell pellet was cryopreserved in liquid nitrogen [8].

### Ethical considerations

The study protocol was approved by the National Mozambican Ethics Review Committee and the Hospital Clínic of Barcelona Ethics Review Committee. All patients were included into the study after written informed consent was given by them or their parents/guardians and were treated following national guidelines of Mozambique or Spain at the time of the study.

### Parasite densities and *msp1*/*msp2* genotyping

Total genomic DNA was extracted from filter papers using QIAmp DNA Mini Kit (Qiagen). Parasitemia was measured by real-time quantitative PCR (qPCR) targeting the *P*. *falciparum* 18S ribosomal RNA gene [69]. The number of concurrent infections (multiplicity of infection, MOI) was estimated as the highest number of *msp-1* or *msp-2* alleles detected in the sample by nested-PCR genotyping [70].

### *var/DC* transcriptional profile

Total RNA prepared in Trizol reagent was extracted using PureLink Micro-to-Midi RNA purification kit (Ambion). RNA was treated with DNaseI (Invitrogen) for 1.5h at 37°C. After discarding the presence of gDNA by PCR-based amplification of *P*. *falciparum* tubulin (PF10_0084) [41] or Seryl-tRNA synthetase genes (*PF*07_0073) [71], reverse transcription was performed using the Super Script III First Strand synthesis system (Invitrogen) with random hexamers primers. Complementary DNA (cDNA) synthesis was confirmed by PCR-based amplification of *P*. *falciparum* tubulin or seryl-tRNA synthetase genes. Then, the transcript levels of *var* subgroups was determined by qPCR using degenerated primers targeting *varA*-exon2, *varB* group (varB-UTR region), *varC* group (varC-UTR region) [28], *varA*-DBLα1 (varA-notDC3) [34] and *var*2CSA (DBL3X domain) [41]. DC transcript levels were assessed by qPCR using a set of primers targeting semi-conserved domains belonging to DC8 (CIDRα1.1), DC9 (DBLγ), DC11 (CIDRβ2+DBLγ7; Forward: TTRGTHACAGCAAAATAYGAAGGTG and reverse: CTCTTACRATATCWCCTATATCKGCA), DC13 (CIDRα1.4), DC16 (CIDRδ) and DC19 (DBLα0.16) [34]. Seryl-tRNA-synthetase gene was used as the reference gene [71]. Individual 20 μL qPCR reactions were performed in duplicate using ABI Prism 7500 Real-Time system (Applied Biosystems) containing 10 μL of Power SYBR Green Master Mix (Applied Biosystems), 4 μL of cDNA and primer concentration of 1μM with cycling conditions of 50°C for 2 min, 95°C for 10 min followed by 40 cycles at 95°C for 15 s and 60°C for 1 min. Data were analyzed using the 7500 System SDS software v1.4. PCR efficiencies of each primer pair were calculated on a standard curve from 7 log dilutions of *P*. *falciparum* 3D7 or *P*. *falciparum* ItG gDNA by the formula (E = 10^−1/m^), where *m* is the slope. Specificity of amplification was assessed by melting-curve analysis of final products. Non-template controls were tested in every plate. Samples with fluorescence detected over the 40 cycles were considered positive. If Ct value felt out of the linear range of the standard curve, a Ct value of 41 was assigned. Ct values were converted to copy numbers [72] using the formula *C/E* ^*ΔCt*^, where *E* is the efficiency of the PCR, *C* is the number of copies of the gene in the *P*. *falciparum* 3D7 or ItG genome [34,72] and ΔCt is the difference in Ct values between a sample and *P*. *falciparum* 3D7 or ItG reference gDNA loaded in each plate [73]. Relative copy number of target genes was calculated by dividing the target gene copies by Seryl-tRNA synthetase gene copies. Transcript levels of *var/DCs* were considered as high if the copy number was ≥0.5-fold of Seryl-tRNA-synthetase copy numbers and low if copy number was <0.5-fold.

### Cytoadhesion profiling

Adhesion of *P*. *falciparum* pediatric isolates to gC1qR (Creative BioMart), CD36, ICAM-1 (R&D Systems) and Duffy-Fc [74], as well as rosetting and PM-agglutination was assessed as previously described [8] and expressed as IEs/mm^2^, percentage of IEs forming rosettes and percentage of IEs in a clump, respectively. Adhesion to purified receptors was considered positive if the number of IEs bound per mm^2^ was higher than the mean binding plus 2 standard deviations to Duffy-Fc coated Petri dishes (19.5 IE/mm^2^) [8], rosetting if frequency of rosettes was higher than 2% [75] and PM-agglutination if frequency of clumps was higher in presence of platelets than in buffer-control [8].

### Antigenic profiling of infected erythrocytes

Forty five *P*. *falciparum* isolates were tested for IgG recognition by plasma from 50 children with SM and 50 with UM, as well as 22 adults recruited in the same study area [8]. After thawing and washing erythrocytes in incomplete RPMI 1640 medium, parasites were matured at 37°C for 18–36 hours until late-stages. Fifty μL of plasmas at 1/10 dilution, previously depleted of antibodies reacting against uninfected A/B-erythrocytes, were mixed with 50 μL of erythrocyte suspension at 1% hematocrit and 0.5–2.2% parasitemia in PBS-1% BSA for 1 hour at room temperature. After sequential incubations with 100 μL of polyclonal rabbit anti-human IgG (DakoCytomation; 1/200 dilution) and 100 μL of Alexa Fluor 488-conjugated donkey anti-rabbit IgG (Invitrogen; 1/1,000) plus 10 μg/mL of ethidium bromide, data from 1,000 ethidium bromide positive events were acquired with a Becton Dickinson LSR Fortessa flow cytometer. Reactivity against IEs was expressed as the difference between the geometric mean fluorescence intensity (GMFI) of IEs and the GMFI of uninfected erythrocytes. A pool of plasma samples from immune Mozambican adults and six plasma samples from non-exposed European adults were included as positive and negative controls, respectively. To allow comparability between isolates, GMFI values from each parasite/plasma combination were scored in relation to the threshold of positivity for each isolate defined as the GMFI of negative controls plus two standard deviations (cut-off). A score of 0 was assigned if GMFI values were below the cut-off; 1 if the value was between one- and two-fold the cut-off; 2 if the value was between two- and three-fold the cut-off; and so on until a maximum score of 5. Breadth of IgG recognition (BoR) was calculated as the sum of scores obtained for each parasite and expressed as percentage of the maximum score possible.

### *var* profiling of *P*. *falciparum* 3D7 selected for cytoadhesion to gC1qR

To select for binding to gC1qR, a *P*. *falciparum* 3D7 culture synchronized in trophozoite/schizont stages was incubated for 1 h in bacteriological Petri plates coated with 2 mL of recombinant gC1qR diluted in PBS (50 μg/mL) [14]. Unbound parasites were collected using a pipette and separated from bound parasites. Both unbound and bound parasites were cultured, with the latter being subjected to a second round of selection for binding to gC1qR. After a limiting dilution cloning, a selected and unselected clone were expanded and tested for binding to gC1qR, CD36, ICAM-1, CSA (Chondroitin sulfate A sodium salt from bovine trachea Sigma-Aldrich) and BSA (Bovine Serum Albumin, Santa Cruz Biotechnology), following standard procedures [8]. The *var* genes transcription profile was determined for both clones by individual qPCR performed in duplicate using primers covering the *P*. *falciparum* 3D7 *var* gene repertoire [71,76].

### Binding between recombinant PfEMP1 domains and gC1qR

Recombinant PFD0020c domains produced in insect or *Escherichia coli* cells [13] were screened for binding against recombinant human EPCR or gC1qR by ELISA (CIDRα1.1, DBLβ12, DBLγ6, DBLγ11, CIDRγ8) and Luminex (DBLα1.2, CIDRα1.1, DBLβ12, DBLγ6, DBLγ11, DBLδ1 and CIDRγ8) in duplicate. For the ELISA assays, MaxiSorp immunoplates (Nunc) were coated overnight at 4°C with 50 μL per well of recombinant human EPCR and gC1qR at 3 μg/mL in PBS pH 7.4. After blocking with PBS 3%-skimmed milk and washing three times with PBS-0.05% TweenR20, PFD0020c domains were added at a concentration of 5 μg/mL in PBS 1%-skimmed milk and incubated for 1 h at 37°C. Secondary anti-V5-HRP antibody diluted in PBS 1%-skimmed milk at 1:3000 was added to each well and incubated for 1 hour at room temperature with gentle shaking. Plates were developed using 100 μL per well of a phosphate solution with o-phenylenediamine. The colorimetric reaction was stopped with 100 μL of 3 M H_2_SO_4_ after 10 minutes and the optical density (OD) was measured at 490 nm. For the Luminex assays, gC1qR was coupled at 50 μg/10^7^ beads to MagPlex-C magnetic carboxylated microspheres (Luminex Corporation) following manufacturer’s instructions. Two thousand coupled beads were incubated with the recombinant PFD0020c domains (DBLα1.2, CIDRα1.1, DBLβ12, DBLγ6, DBLγ11, DBLδ1 and CIDRγ8) at 1ug/ml in incubation buffer (IB; 1% Skim Milk in PBS), overnight at 4°C. After 3 washes with washing buffer (PBS + 0.5% Tween20 + 0.25% skim milk), the beads were incubated with anti-V5 from mouse (ThermoFisher, R960-25) at 1/2500 in IB at room temperature for 1 hour, followed by an incubation with anti-mouse biotin conjugated antibody (Sigma, B7401) at 1/10000 in IB for 1 hour at RT, and streptavidin-R-phycoerythrin (Sigma, 42280) at 1/1000 in IB for 30 minutes at RT, with 3 washes after each incubation. Median Fluorescence Intensity was obtained using the Luminex 100/200 System (Luminex Corp., Austin, Texas).

### Inhibition of *P*. *falciparum-*infected erythrocyte binding to gC1qR and Human Brain Microvascular Endothelial Cells

Anti-sera against domains belonging to *PF*D0020c (α-CIDRα1.1_PFD0020c_, α-DBLβ12_PFD0020c_, α-DBLγ6_PFD0020c_, α-CIDRγ8_PFD0020c)_), *PF*08_0140 (α-DBLβ12_PF08_0140_) and PFI1820w (α-PFI1820w) were produced in rabbit [13]. After depleting rabbit sera of antibodies against human erythrocytes, IgGs were purified by Affi-Gel Protein-A MAPS II Kit (Bio-Rad, Richmond, CA) and quantified using EPOCH spectrophotometer. To test their ability to inhibit binding of *P*. *falciparum* to recombinant gC1qR, 20 μL pellet of *P*. *falciparum* 3D7 pigmented trophozoite (≥2% parasitaemia, 1% hematocrit) were incubated in duplicate for 1.5 h at 37°C with 300 μg/mL rabbit IgGs diluted in PBS and used for a standard adhesion assay in Petri dishes [8]. Similar procedures were used to test inhibition of gC1R binding by 4 Mozambican *P*. *falciparum* isolates (*Pf*moz 1–4).

Human Brain Microvascular Endothelial Cells (HBMEC; Innoprot) were seeded on flat-bottomed Nunclon Δ Surface (Nunc cat number: 150628) 12-well plates 3 to 4 days before assays and allowed to growth to 30–40% confluence in endothelial cell medium (Innoprot). Prior to the adhesion assay, HBMECs were washed once with PBS followed by addition of 20 μl 2% FCS in RPMI/well. For binding inhibition, IgG-purified anti-PfEMP1 rabbit antibodies and PBS alone were added to 2% parasitemia and 2% hematocrit late-stage IEs at a final concentration of 300 μg/ml incubated for 1.5 h at 37°C. 300 μl of the IE suspension were added to each well and co-incubated on a rocking table for 1 hour at room temperature. Unbound infected erythrocytes were removed by several gentle washes. Wells were then fixed in 2% glutaraldehyde over night at room temperature and stained with Giemsa for 10 min. Binding was quantified by determining the number of IEs adhering per endothelial cells nuclei in 50 random fields under 400× magnification. All binding assays were done in triplicate. The percentage of binding was expressed relative to binding in the absence of antibodies.

### IgG measurement in human plasma samples

IgG reactivity against the recombinant PFD0020c domains (DBLα1.2, CIDRα1.1, DBLβ12, DBLγ6, DBLγ11, DBLδ1 and CIDRγ8) was assessed in 135 malaria-infected Mozambican children (67 with severe malaria and 68 with uncomplicated malaria) and 18 Spanish adults never exposed to malaria. PfEMP1 domains or BSA (Sigma, A7030, as background control) were coupled at 50 μg/10^7^ beads to MagPlex-C magnetic carboxylated microspheres (Luminex Corporation) following manufacturer’s instructions. Multiplexed beads were incubated with plasma samples (1/50 dilution) and antibody levels were detected as described elsewhere [77]. Positive, negative and background controls were added to each plate. Median Fluorescence Intensity (MFI) was obtained from the InVitrogen Luminex platform (xPONENT Software, at least 100 counts/analyte) and normalized for inter-plate variability by multiplying MFIs by the median value of a positive control from all plates and dividing by each plate’ value.

### Statistical analysis

Correlations between variables were assessed by Spearman’s rank coefficient, with Benjamini-Hochberg correction for multiple comparisons. Continuous data were compared between matched case/control pairs by Sign-test and between non-paired groups by Mann-Whitney test. BoR was compared between groups by a Test for trend across ordered groups and between isolates transcribing *var/DCs* at low- or high-levels by negative binomial regression models adjusted by age. Mean ratio of IgGs and 95% confidence intervals between Mozambican children and Spanish adults, as well as between Mozambican children older than 2.5 years of age and less than 2.5 years were calculated in linear regression models, with log-transformed MFIs. Statistical analysis was performed with Stata/SE software (version 12.0; StataCorp).

## Supporting Information

S1 Text Table A. Percentage of isolates from Mozambican children (n = 86) showing cytoadherence, and cytoadherence levels. Table B. Prevalence of isolates expressing the target var/DC genes tested. Table C. Transcript levels of *var/DCs* by severe malaria symptoms. Fig A. Binding of recombinant PFD0020c domains (DBLα1.2, CIDRα1.1, DBLβ12, DBLγ6, DBLγ11, DBLδ1 and CIDRγ8) to gC1qR as assessed by Luminex assay. Fig B. Percentage of inhibition of *P*. *falciparum* cytoadhesion by purified antibodies against PFD0020c domains to EPCR or CD36 receptors. Fig C. IgG recognition of PFD0020c domains by plasmas from malaria-infected Mozambican children and never-exposed individuals from Spain.
(DOCX)
