# Supplementary material for: Cytoadhesion to gC1qR through Plasmodium falciparum Erythrocyte Membrane Protein 1 in Severe Malaria
Source: PLoS Pathog. 2016 Nov 11;12(11):e1006011. doi: 10.1371/journal.ppat.1006011 (PMC5106025; doi:10.1371/journal.ppat.1006011)
Supplement: S1 Text — Table A. Percentage of isolates from Mozambican children (n = 86) showing cytoadherence, and cytoadherence levels. Table B. Prevalence of isolates expressing the target var/DC genes tested. Table C. Transcript levels of var/DCs by severe malaria symptoms. Fig A. Binding of recombinant PFD0020c domains (DBLα1.2, CIDRα1.1, DBLβ12, DBLγ6, DBLγ11, DBLδ1 and CIDRγ8) to gC1qR as assessed by Luminex assay. Fig B. Percentage of inhibition of P. falciparum cytoadhesion by purified antibodies against PFD0020c domains to EPCR or CD36 receptors. Fig C. IgG recognition of PFD0020c domains by plasmas from malaria-infected Mozambican children and never-exposed individuals from Spain. (DOCX) [file ppat.1006011.s001.docx]

Cytoadhesion to gC1qR through *Plasmodium falciparum* erythrocyte membrane protein 1 in severe malaria

# Supporting information

## **Figure A.** Binding of recombinant PFD0020c domains (DBLα1.2, CIDRα1.1, DBLβ12, DBLγ6, DBLγ11, DBLδ1 and CIDRγ8) to gC1qR as assessed by Luminex assay.

Beads coupled with gC1qR were incubated with recombinant domains and binding was assessed by detection with anti-V5 from mouse and anti-mouse biotin conjugated antibody. Median Fluorescence Intensity (MFI) obtained using the Luminex® 100/200™ System (Luminex Corp., Austin, Texas) expresses the level of binding to gC1qR (mean and standard error of the mean).

##
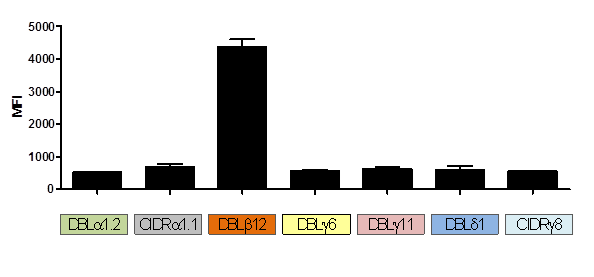


## **Figure B.** Percentage of inhibition of *P. falciparum* cytoadhesion by purified antibodies against PFD0020c domains to EPCR or CD36 receptors

**A)** CD36 binding levels in absence of antibodies was 87 IE/mm^2^ (SD 6) for *Pf*moz1*;* 80 IE/mm^2^ (SD 10) for *Pf*moz2; 88 IE/mm^2^ (SD 7) for *Pf*moz3 and 90 IE/mm^2^ (SD 3) for *Pf*moz4. **B)** EPCR binding levels in absence of antibodies was 127 IE/mm^2^ (SD 9) for *Pf*moz1*;* 50 IE/mm^2^ (SD 10) for *Pf*moz2; 36 IE/mm^2^ (SD 6) for *Pf*moz3 and 120 IE/mm^2^ (SD 6) for *Pf*moz4. **C)** DC8 transcript levels in isolates tested for inhibition of adhesion to gC1qR, targeted by primers previously reported [[34](#_ENREF_34)] Transcript levels (y axis) correspond to relative copy number relative to seryl-tRNA synthetase gene copies (X100). Binding is expressed as the percentage of mean binding in absence of antibodies. Bars represent the mean and standard deviation, with *** indicating *P*<0.001.


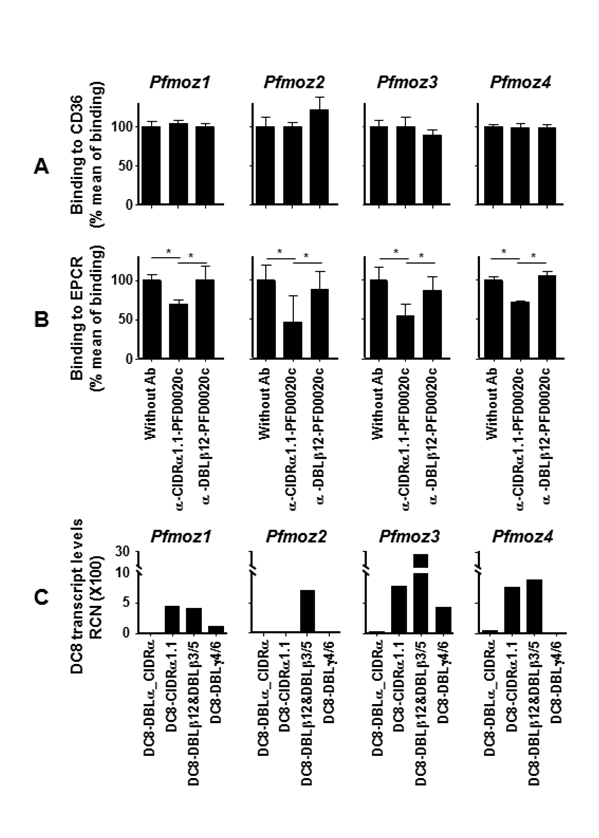


## **Figure C.** IgG recognition of PFD0020c domains by plasmas from malaria-infected Mozambican children and never-exposed individuals from Spain.

**A)** Mean ratio of IgGs and 95% confidence intervals between Mozambican children (n=135) and Spanish adults (n=18=); **B)** Mean ratio of IgGs and 95% confidence intervals between Mozambican children older than 2.5 years of age (n=68) and less than 2.5 years (n=71); **C)** MFI levels obtained from plasmas of Mozambican children and Spanish adults.


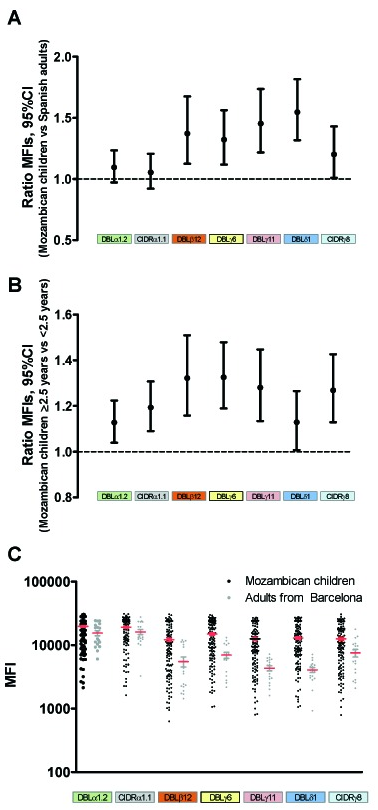


## SUPPLEMENTARY TABLES

## **Table A. Percentage of isolates from Mozambican children (n=86) showing cytoadherence and cytoadherence levels.**

|  | n | % | Median | IQR |
| --- | --- | --- | --- | --- |
| **CD36** | 76 | 88 | 180 | 101-353 |
| **gC1qR** | 38 | 44 | 60 | 45-155 |
| **ICAM1** | 37 | 43 | 55 | 39-105 |
| **PM-agg** | 57 | 66 | 7% | 2-22 |
| **Rosetting** | 31 | 36 | 2% | 1-5 |

## **Table B. Prevalence of isolates expressing the target *var*/DC genes tested.**

|  |  |  | **Spain** |  |  |  | **Mozambique** |  |  |
| --- | --- | --- | --- | --- | --- | --- | --- | --- | --- |
|  | **All (N=132)** |  | **Travellers (N=21)** |  | **SM (N=43)** |  | **UM (N=43)** |  | **Adults (N=25)** |
|  | **n (%)** |  | **n (%)** |  | **n (%)** |  | **n (%)** |  | **n (%)** |
| varA-exon2 | 132 (100) |  | 21 (100) |  | 43 (100) |  | 43 (100) |  | 25 (100) |
| varA-DBLa1-notDC3 | 130 (98) |  | 21 (100) |  | 43 (100) |  | 42 (98) |  | 24 (96) |
| varB-UpsB | 127 (96) |  | 17 (81) |  | 43 (100) |  | 43 (100) |  | 24 (96) |
| varC-UpsC | 129 (98) |  | 21 (100) |  | 43 (100) |  | 42 (98) |  | 23 (92) |
| varE-DBL3X | 128 (97) |  | 20 (95) |  | 43 (100) |  | 42 (98) |  | 23 (92) |
| DC8-CIDRa1.1 | 112 (85) |  | 15 (71) |  | 39 (91) |  | 41 (95) |  | 17 (68) |
| DC9-DBLg | 113 (86) |  | 18 (86) |  | 38 (88) |  | 38 (88) |  | 19 (76) |
| DC11-CIDRb2+DBLg7 | 132 (100) |  | 21 (100) |  | 43 (100) |  | 43 (100) |  | 25 (100) |
| DC13-CIDRa1.4 | 52 (39) |  | 8 (38) |  | 18 (42) |  | 17 (40) |  | 9 (36) |
| DC16-CIDRd | 105 (80) |  | 17 (81) |  | 34 (79) |  | 37 (86) |  | 17 (68) |
| DC19-DBLa0.16 | 115 (87) |  | 17 (81) |  | 40 (93) |  | 37 (86) |  | 21 (84) |

SM: severe malaria; UM: uncomplicated malaria.

## **Table C**. Transcript levels of *var/DCs* by severe malaria symptoms.

Transcript levels correspond to relative copy number relative to seryl-tRNA synthetase gene copies (X100). Data presented correspond to median and interquartile range (IQR) of uncomplicated malaria (UM) and severe malaria cases (SM) as well as for the difference (SM-UM) of matched cases (MC). Transcript levels were compared between matched case/control pairs by Sign-test, with * indicating P≤0.05. DC: Domain Cassette; SAn: Severe Anemia; Pro: Prostration; ARD: Acidosis/Respiratory Distress; MSz: Multiple Seizures.
